# Transcriptome Profiling Reveals Genetic Basis of Muscle Development and Meat Quality Traits in Chinese Congjiang Xiang and Landrace Pigs

**DOI:** 10.3390/metabo15070426

**Published:** 2025-06-22

**Authors:** Jiada Yang, Qiaowen Tang, Chunying Sun, Qiuyue Li, Xiaoyu Li, Lu Hou, Yi Yang, Kang Yang

**Affiliations:** 1 School of Life and Health Science, Kaili University, Kaili 556011, China; yangjiada2@163.com (J.Y.); 19885073498@163.com (Q.T.); 13595520703@163.com (C.S.); 18185585150@163.com (Q.L.); 19315423801@163.com (X.L.); houlukluniv@163.com (L.H.); 2 Institute of Animal Nutrition and Feed Science, College of Animal Sciences, Guizhou University, Guiyang 550025, China; yyang23@gzu.edu.cn

**Keywords:** transcriptome, pig, *longissimus dorsi*, meat quality, *PPAR* signaling

## Abstract

(1) Objectives: Understanding the genetic basis of muscle development and meat quality traits in divergent pig breeds is crucial for advancing precision breeding strategies. (2) Methods: This study investigated transcriptome differences in the *longissimus dorsi* muscle between Chinese Congjiang Xiang (CX) and Landrace (LAN) pigs. RNA sequencing was performed on muscle tissues from ten individuals of each breed, generating 874.5 million raw reads with an average mapping rate of 89.3% to the pig reference genome. (3) Results: Transcriptional profiling revealed distinct expression patterns with 785 genes exclusively expressed in CX pigs and 457 genes unique to LAN pigs, while 7099 co-expressed genes were shared by both breeds. Differential expression analysis identified 2459 significantly different genes (|log2FC| ≥ 1, adjusted *p*-value < 0.05), with 1745 up-regulated and 714 down-regulated in CX pigs. Among the most significantly up-regulated genes in CX pigs were flavor-associated genes (*ELOVL5/6*, *FASN*, *DGAT2*, *ALDH1A3*, *PPAR-γ*) with log2FC values ranging from 1.21 to 3.88. GO and KEGG pathway analyses revealed that up-regulated genes in CX pigs were significantly enriched in immune response pathways (adjusted *p*-value < 0.01), while down-regulated genes were primarily associated with myosin complex formation and *PPAR* signaling pathway. PPI network analysis identified *PPAR-γ* as a central hub gene with 16 direct interactions to other flavor-related genes. (4) Conclusions: These findings demonstrate that the superior meat flavor characteristics of indigenous Chinese pigs are driven by enhanced expression of lipid metabolism genes and distinctive immune-related pathways, providing specific molecular targets for breeding programs aimed at improving meat quality while maintaining production efficiency in commercial breeds.

## 1. Introduction

According to statistics from the Food and Agriculture Organization of the United Nations (FAO) in 2024, pork accounts for the largest share of global meat consumption, about 36% [1], followed by poultry meat, about 35%, while beef and mutton account for about 22% and 5%, respectively, and other meat varieties account for about 2% of the market share. This pork popularity stems from its nutritional profile, which includes high-quality proteins, essential amino acids, and various micronutrients crucial for human health and development [2]. In recent years, meat characteristics, particularly muscle quality traits such as intramuscular fat content, tenderness, and water-holding capacity, have emerged as significant factors influencing consumer purchasing decisions [3]. The *longissimus dorsi*, commonly known as the loin muscle, is especially valued for its tenderness and flavor attributes, making it one of the most economically important cuts in pork production [4]. These quality traits are predominantly influenced by three major factors: genetics, nutrition, and environmental conditions [5]. Among these, genetic factors play a fundamental role in determining muscle development, fat deposition patterns, and ultimately, meat quality characteristics, contributing to approximately 30–45% of the variation observed in key traits such as intramuscular fat content [6].

With the advancement of modern molecular breeding techniques, the improvement of pork quality has entered a new era that extends beyond traditional selective breeding methods. Contemporary approaches integrate conventional breeding with molecular biology, bioinformatics, and computational technologies to achieve more precise and efficient genetic improvements. Molecular marker-assisted selection, genomic selection, and gene editing technologies have revolutionized pig breeding by enabling the identification and utilization of genes directly associated with economically important traits [7,8]. The screening and validation of functional genes have become crucial components of molecular breeding programs aimed at enhancing meat quality characteristics [9]. Particularly, the genetic mechanisms governing fat deposition in muscle tissues involve intricate and highly synchronized gene expression programs that regulate adipogenesis, lipid metabolism, and fatty acid composition. These complex biological processes ultimately determine the sensory qualities of pork, including juiciness, tenderness, and flavor intensity, making them priority targets for genetic improvement efforts in pig breeding [10].

Transcriptomics has emerged as a pivotal molecular technology in biological research, offering comprehensive insights into gene expression patterns under various physiological and developmental conditions. The rapid advancement of RNA sequencing (RNA-Seq) technology has significantly expanded its application in the livestock industry, providing unprecedented resolution in transcriptome profiling [11]. RNA-Seq analyses enable the identification of differentially expressed genes, novel transcripts, alternative splicing events, and regulatory networks across different species, breeds, and tissues. In pig research specifically, RNA-Seq has been extensively employed to investigate transcriptional variations associated with muscle development, fat deposition, and meat quality traits [12,13]. For example, RNA-Seq analysis identified key genes affecting intramuscular fat content in Xidu black pig, Diannan small ears pig, Wujin pig, and Landrace (LAN) pig [14]. These studies have identified numerous candidate genes and molecular pathways involved in muscle fiber formation, adipogenesis, and lipid metabolism, contributing substantially to our understanding of the genetic basis of pork quality. The application of transcriptomics is particularly valuable for comparing divergent pig breeds with distinct phenotypic characteristics, as it can reveal the underlying genetic mechanisms responsible for their phenotypic differences [15].

The Chinese Congjiang Xiang (CX) pig, indigenous to Congjiang County in Guizhou Province, represents one of China’s valuable native pig genetic resources (Figure S1). This breed has evolved over centuries under local environmental conditions and selective pressures, developing unique adaptations to the mountainous terrain and subtropical climate of southwestern China. CX pigs are characterized by their small body size, black coat color, and exceptional adaptability to extensive farming systems with limited resources. Their notable resilience to diseases, stress tolerance, and reproductive efficiency highlight their evolutionary advantages as a local breed [16]. Most significantly, CX pigs exhibit distinctive meat quality characteristics, including a higher intramuscular fat content (typically 3–5%), finer muscle fibers, and a distinctive flavor profile compared to commercial breeds [17]. These attributes contribute to the exceptional tenderness, juiciness, and unique taste of their meat, which is highly valued in traditional Chinese cuisine and local gastronomy. The Landrace (LAN) pig, originating from Denmark and subsequently improved in various European countries, represents one of the most widely utilized commercial pig breeds globally. They demonstrate superior feed conversion efficiency and lean meat percentage (typically 58–62%) compared to most indigenous Chinese breeds [18]. However, the intensive selection for production efficiency in LAN pigs has been associated with reduced intramuscular fat content (generally 1.5–2.5%), which may affect certain aspects of meat quality such as flavor intensity and juiciness [19]. The comparative analysis of muscle traits between CX and LAN pigs reveals distinct differences in muscle fiber composition, fat distribution patterns, and metabolic characteristics that directly influence their respective meat quality profiles.

The selection of CX and LAN pigs as research models provides an exceptional opportunity to investigate the genetic basis of divergent muscle development and quality traits. These breeds represent distinct evolutionary paths in pig domestication and improvement: CX pig embodies centuries of natural selection and adaptation to local conditions with minimal human intervention, while LAN pig exemplifies intensive artificial selection for production efficiency and carcass traits [20]. This genetic divergence has resulted in pronounced phenotypic differences in muscle characteristics, particularly in intramuscular fat content, muscle fiber composition, and metabolic properties. Comparing these genetically distant breeds enables the identification of key genes, regulatory elements, and biological pathways associated with critical muscle quality traits [21]. Additionally, the contrasting fat deposition patterns between these breeds—with CX demonstrating superior marbling capacity despite lower overall growth rates—presents a valuable model for understanding the molecular mechanisms governing intramuscular adipogenesis [22]. The knowledge derived from studying these contrasting breeds has significant potential applications for precision breeding strategies aimed at improving meat quality while maintaining production efficiency in commercial pig lines.

In the present study, we employed RNA-Seq technology to analyze differential gene expression patterns in the *longissimus dorsi* muscle of Chinese CX and LAN pigs. This comprehensive transcriptomic approach allowed us to identify and characterize differentially expressed genes (DEGs) between these breeds with distinct muscle development and quality characteristics. Furthermore, this study contributes to the broader understanding of the genetic architecture governing muscle development in divergent pig breeds and provides valuable resources for future molecular breeding strategies aimed at optimizing meat quality traits in pigs.

## 2. Materials and Methods

### 2.1. Animals and Sample Collection

Ten male pigs each from Congjiang Xiang (CX) and Landrace (LAN) breeds were used in this study. This sample size is comparable to similar transcriptome studies in livestock genetics [23]. The pigs were raised under the same similar altitudes and shared similar natural climatic conditions in Congjiang County, Qiandongnan Miao and Dong Autonomous Prefecture, Guizhou Province. All animals were fed cooked food (mainly consisting of corn, bean curd residue, rice bran, fruits, and vegetables) throughout the study period with *ad libitum* access to feed and water. All animals (all males) were slaughtered with electrical stunning followed by exsanguination at 12 months of age, with an average body weight of 74.95 ± 18.81 kg (CX, normal weight range: 60–90 kg) and 189.95 ± 40.95 kg (LAN, normal weight range: 150–220 kg). The *longissimus dorsi* (LD) muscle tissues were immediately collected 45 min postmortem from the left side of the carcass between the 12th and 13th ribs, snap-frozen in liquid nitrogen, and stored at −80 °C until RNA extraction. All the animal experiments in the present study strictly complied with the relevant regulations regarding the care and use of experimental animals issued by the Academic Committee of Kaili University (Approval ID: 202402).

### 2.2. RNA Extraction, Library Construction, and Sequencing

Total RNA was isolated using TRIzol reagent (Invitrogen, CarIsbad, CA, USA) according to the manufacturer’s protocol. The acquisition of mRNA utilizes the structural feature that most mRNAs in eukaryotes have a polyA tail, and the mRNA with a polyA tail is enriched by Oligo(dT) magnetic beads. RNA purity and concentration were assessed using a NanoDrop ND-2000 spectrophotometer (NanoDrop Technologies, Wilmington, DE, USA), and RNA integrity was checked by 1% agarose gel electrophoresis. The RNA integrity number (RIN) was determined using an Agilent 2100 Bioanalyzer (Agilent Technologies, Santa Clara, CA, USA), and only samples with RIN ≥ 7.5 were used for library construction.

Sequencing libraries were generated using the TruSeq RNA Sample Preparation Kit (Illumina, San Diego, CA, USA) following the manufacturer’s instructions. Briefly, mRNA was purified from total RNA using oligo(dT) magnetic beads. The mRNA was then fragmented and used for first-strand cDNA synthesis with random hexamer primers and reverse transcriptase. Second-strand cDNA synthesis was subsequently performed using DNA polymerase I and RNase H. The double-stranded cDNA was purified and subjected to end repair, A-tailing, and adapter ligation. After size selection using AMPure XP beads (Beckman Coulter, Brea, CA, USA), the libraries were PCR-amplified and purified. Library quality was assessed on the Agilent Bioanalyzer 2100 system. The clustering of the index-coded samples was performed on a cBot Cluster Generation System using TruSeq PE Cluster Kit v3-cBot-HS (Illumina, USA). The libraries were sequenced on an Illumina NovaSeq 6000 platform, and 150 bp paired-end reads were generated.

### 2.3. Read Mapping and Differential Expression Analysis

Raw reads in FASTQ format were first processed using Trimmomatic (v0.36) to remove adapter sequences, low-quality reads (Q < 20), and short reads (<50 bp). The cleaned reads were then aligned to the pig reference genome (Sscrofa 11.1) using HISAT2 (v2.1.0) with default parameters. StringTie (v1.3.5) was used to calculate the fragments per kilobase of transcript per million mapped reads (FPKM) value for each gene. Differential expression analysis between CX and LAN pigs was performed using the DESeq2 R package (v1.24.0).

### 2.4. Functional Enrichment and Pathway Analysis

Gene Ontology (GO) and Kyoto Encyclopedia of Genes and Genomes (KEGG) pathway enrichment analyses of differentially expressed genes (DEGs) were performed using the clusterProfiler R package (v3.12.0). GO terms and KEGG pathways with adjusted *p*-value < 0.05 were considered significantly enriched.

### 2.5. Protein–Protein Interaction (PPI) Network Analysis

The PPI network for DEGs was constructed using the STRING database (v11.0) with a confidence score ≥ 0.9. Cytoscape (v3.7.2) was used to visualize the PPI network.

### 2.6. Validation by Quantitative Real-Time PCR (qRT-PCR)

Ten DEGs (five up-regulated and five down-regulated in CX pigs) were randomly selected for qRT-PCR validation. The qRT-PCR primers (Table S1) were designed using Primer Premier 5.0 software. The qRT-PCR analysis was performed on a LightCycler 480 II Real-time PCR System (Roche, Basel, Switzerland) using TB Green Premix Ex Taq II (Takara, Osaka, Japan) according to the manufacturer’s instructions. The porcine GAPDH gene was used as an internal control. The relative expression levels of the selected DEGs were calculated using the 2−ΔΔCt method.

### 2.7. Statistical Analysis

Genes with |log2(fold change)| ≥ 1 and adjusted *p*-value < 0.05 were considered as DEGs. Ten biological replicates per breed with three technical replicates for each were used for qRT-PCR validation. The relative expression levels of the selected DEGs were performed using Student’s *t*-test in SPSS 22.0 software (IBM, Armonk, NY, USA), and a *p*-value < 0.05 was considered statistically significant.

## 3. Results

### 3.1. Overview of RNA Sequencing Data

**Figure 1:**
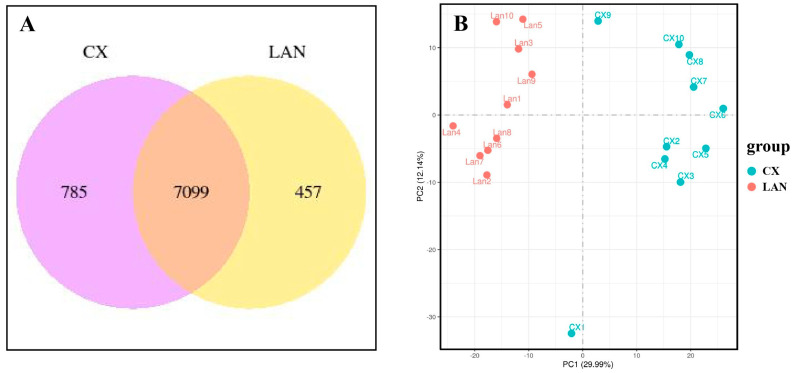
Transcriptome Comparison of CX and LAN Pigs: (**A**) Venn diagram illustrating the distribution of expressed genes in longissimus dorsi muscle between Congjiang Xiang (CX) and Landrace (LAN) pigs. (**B**) Principal component analysis (PCA) plot based on global gene expression profiles of all samples (n = 10 per breed). Red dots represent individual CX pig samples, and blue dots represent individual LAN pig samples.

To systematically investigate the transcriptome differences between Congjiang Xiang (CX) and Landrace (LAN) pigs, we performed RNA sequencing on *longissimus dorsi* (LD) muscle tissues from ten individuals of each breed. A total of 874.5 million raw reads were generated, with an average of 43.7 million reads per sample. After quality control, 846.0 million clean reads were obtained and mapped to the pig reference genome (Sscrofa 11.1), with an average mapping rate of 89.3% (Table S2). The co-expression Venn diagram analysis revealed distinct transcriptional profiles between the two porcine breeds, with 785 genes being exclusively expressed in CX pigs and 457 genes uniquely expressed in LAN pigs. Notably, the intersection region demonstrated a core set of 7099 co-expressed genes in the LD muscle shared by both breeds (Figure 1A). Principal component analysis (PCA) based on the expression profiles showed a clear separation between CX and LAN pigs (Figure 1B), indicating distinct transcriptome landscapes in the LD muscle between the two breeds.

### 3.2. Identification of Differentially Expressed Genes

**Figure 2:**
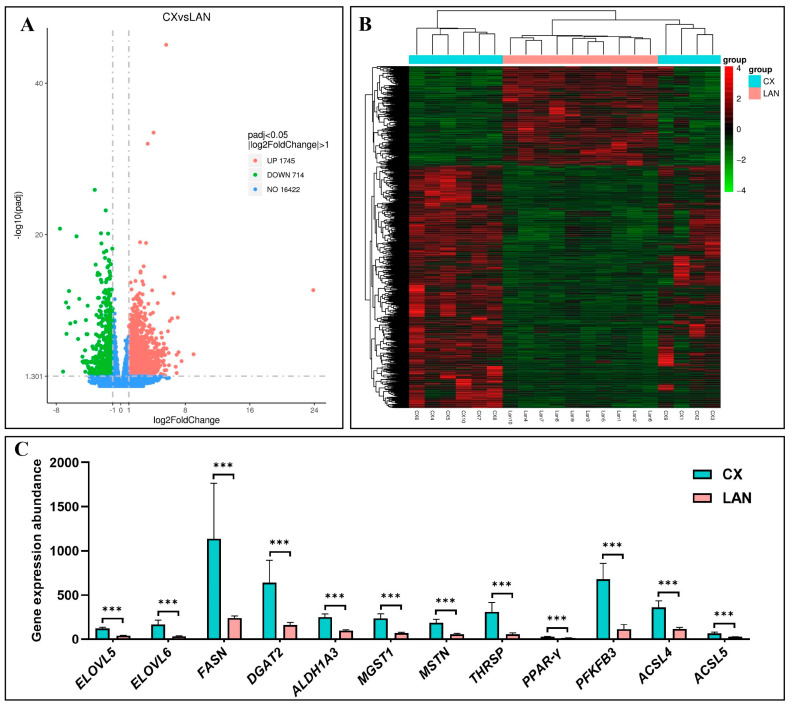
Differentially Expressed Genes (DEGs) Between CX and LAN Pigs: (**A**) Volcano plot displaying the distribution of DEGs between Congjiang Xiang (CX) and Landrace (LAN) pigs. The *x*-axis represents log2 fold change, while the *y*-axis represents −log10(adjusted *p*-value). Red dots indicate significantly up-regulated genes in CX pigs (log2FC ≥ 1, adjusted *p*-value < 0.05), blue dots indicate significantly down-regulated genes in CX pigs (log2FC ≤ −1, adjusted *p*-value < 0.05), and gray dots represent genes with no significant differential expression. (**B**) Hierarchical clustering heatmap of DEGs showing distinct expression patterns between CX and LAN breeds. Each column represents an individual animal sample (n = 10 per breed), and each row represents a differentially expressed gene. Color intensity indicates the level of gene expression, with red representing higher expression and blue representing lower expression. (**C**) Expression levels of key flavor-related genes that were significantly up-regulated in CX pigs compared to LAN pigs. The *y*-axis represents normalized expression values (FPKM) (*** *p*-value < 0.001).

Differential expression analysis identified a total of 2459 differentially expressed genes (DEGs) between CX and LAN pigs, including 1745 up-regulated and 714 down-regulated genes in CX pigs (Figure 2A). Hierarchical clustering analysis revealed that the DEGs could clearly distinguish the two breeds (Figure 2B), suggesting their potential roles in driving the phenotypic differences. The details of the top 10 up- and down-regulated DEGs are shown in Table 1 and Table 2, respectively. Notably, regulatory factors known to be closely associated with meat flavor formation, such as *ELOVL5/6*, *FASN*, *DGAT2*, *ALDH1A3*, *MGST1*, *MSTN*, *THRSP*, *PPAR-γ*, *PFKFB3*, and *ACSL4/5*, were significantly up-regulated in CX pigs (Figure 2C), implying enhanced meat flavor characteristics in this breed.

### 3.3. Functional Enrichment Analysis of DEGs

**Figure 3:**
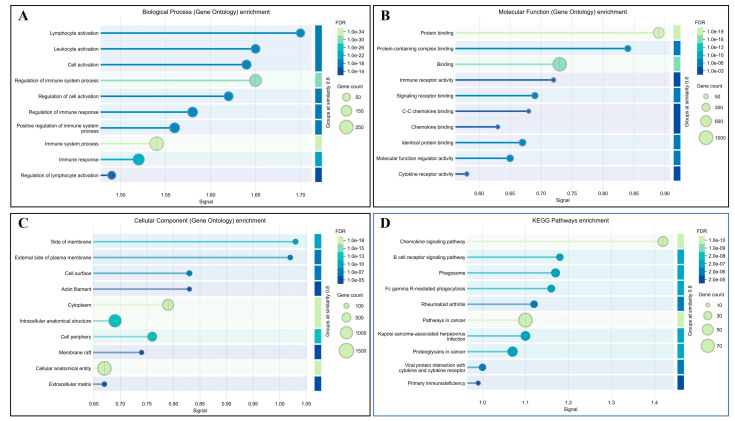
Functional Enrichment Analysis of DEGs Between CX and LAN Pigs: (**A**) Gene Ontology (GO) enrichment analysis of Biological Process (BP) terms for differentially expressed genes. (**B**) GO enrichment analysis of Molecular Function (MF) terms. (**C**) GO enrichment analysis of Cellular Component (CC) terms. (**D**) KEGG pathway enrichment analysis of DEGs. The dot plot illustrates significantly enriched pathways, with dot size representing the number of genes involved and color intensity indicating statistical significance (adjusted *p*-value).

To gain insights into the biological functions of the DEGs, we performed Gene Ontology (GO) and Kyoto Encyclopedia of Genes and Genomes (KEGG) pathway enrichment analyses. GO analysis revealed that the up-regulated genes in CX pigs were significantly enriched in immune response, defense response, regulation of the immune system process, positive regulation of the immune system process, and response to external stimulus (Figure 3A–C). In contrast, the down-regulated genes were mainly involved in myosin complex, proton-transporting V-type ATPase complex, and actin cytoskeleton. KEGG pathway analysis showed that the up-regulated genes were highly enriched in pathways related to viral protein interaction with cytokine and cytokine receptor, hematopoietic cell lineage, chemokine signaling pathway, cytokine–cytokine receptor interaction, and cell adhesion molecules (Figure 3D). The down-regulated genes were significantly associated with *PPAR* signaling pathway and glycerolipid metabolism. These findings may contribute to the superior meat flavor characteristics in CX pigs compared to LAN pigs.

### 3.4. PPI Network Analysis of DEGs

**Figure 4:**
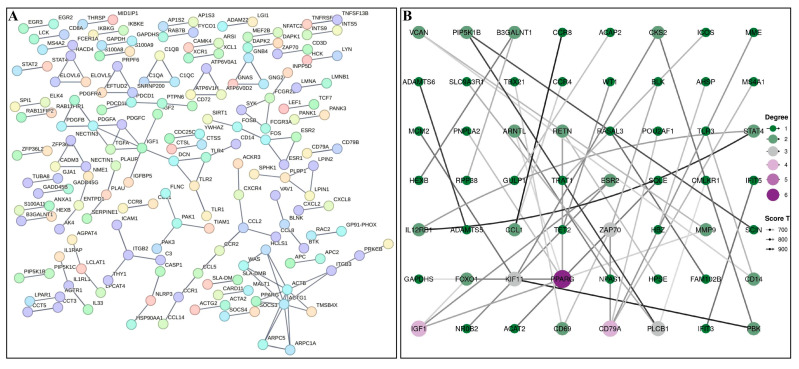
Protein–Protein Interaction Network Analysis of Differentially Expressed Genes: (**A**) Global protein–protein interaction (PPI) network constructed using the STRING database, containing 1306 nodes (proteins) and 138 edges (interactions) derived from differentially expressed genes between Congjiang Xiang (CX) and Landrace (LAN) pigs. Nodes represent individual proteins encoded by DEGs, while edges represent predicted functional associations between proteins. (**B**) Focused subnetwork highlighting PPAR-γ as a central hub gene and its interactions with other flavor-related genes.

To further explore the functional interactions among the DEGs, we constructed a protein–protein interaction (PPI) network using the STRING database. The PPI network contained 1306 nodes and 138 edges (Figure 4A). *PPAR-γ* were identified as hub genes in the network (Figure 4B), indicating their central regulatory roles in meat flavor formation. Moreover, several key genes (e.g., *ACTG1*, *ACTB*, *IGF1*) were also found to be closely connected in the network, suggesting coordinated regulation of these processes.

### 3.5. Validation of DEGs by qRT-PCR

All designed primers demonstrated good amplification efficiency and specificity. The qRT-PCR validation results revealed that the expression patterns of all ten selected genes were highly consistent with the RNA-Seq analysis findings, with consistency rates ranging from 95.9% to 99.6% (Table 3). In CX pigs, *AP1S2*, *FCGR3A*, *SLA-DQA*, *CD37*, and *EMP2* genes were significantly up-regulated, while *VPS72*, *UBL5*, *GDE1*, *AQP7*, and *CUL1* genes were significantly down-regulated (*p* < 0.001). These results confirm the reliability of our transcriptome analysis findings.

## 4. Discussion

In the present study, we performed RNA-Seq analysis to identify differentially expressed genes in the *longissimus dorsi* muscle between Chinese Congjiang Xiang (CX) and Landrace (LAN) pig breeds. Our results revealed substantial transcriptional differences between these breeds, with 2459 differentially expressed genes (DEGs) identified, including 1745 up-regulated and 714 down-regulated genes in CX pigs compared to LAN pigs. Principal component analysis and hierarchical clustering analysis demonstrated distinct transcriptional profiles that clearly separated the two breeds, highlighting the significant genetic divergence between indigenous Chinese and commercial European pig breeds. We observed breed-specific gene expression patterns, with 785 genes exclusively expressed in CX pigs and 457 genes unique to LAN pigs, suggesting their potential contributions to phenotypic differences. Notably, several regulatory factors associated with meat flavor development, including *ELOVL5/6*, *FASN*, *DGAT2*, *ALDH1A3*, *MGST1*, *MSTN*, *THRSP*, *PFKFB3*, and *ACSL4/5*, were significantly up-regulated in CX pigs, which may explain the enhanced flavor characteristics typically associated with this indigenous breed. We identified 16,422 genes that showed similar expression levels in both breeds, representing core muscle functions conserved across the breeds.

The distinct transcriptional profiles observed between CX and LAN pigs provide valuable insights into the molecular basis of breed-specific characteristics. The 785 genes exclusively expressed in CX pigs may represent a unique genetic signature that contributes to the distinctive traits of this indigenous Chinese breed. Indigenous pig breeds have evolved under diverse environmental conditions and selective pressures that differ from those experienced by commercial breeds, leading to genetic adaptations reflected in their transcriptional landscapes. Similar breed-specific expression patterns have been reported in previous studies comparing indigenous and commercial pig breeds. For instance, Yang et al. (2024) identified unique expression patterns in Tibetan pigs that contributed to their adaptation to high-altitude environments [24], while Li et al. (2019) reported breed-specific transcriptional signatures in Meishan pigs associated with their superior fertility traits [25]. Our findings add to this growing body of evidence suggesting that breed-specific gene expression patterns underlie the phenotypic divergence between indigenous and commercial pig breeds, highlighting the value of indigenous genetic resources for understanding the molecular basis of economically important traits.

The up-regulation of flavor-related genes in CX pigs represents one of the most significant findings of our study. Genes involved in lipid metabolism, including ELOVL5/6, FASN, and DGAT2, play crucial roles in the biosynthesis and modification of fatty acids that contribute to meat flavor and tenderness [26]. ELOVL5/6 encode elongases that catalyze the elongation of fatty acids, while FASN is a key enzyme in de novo fatty acid synthesis [27]. DGAT2 catalyzes the final step in triglyceride synthesis, contributing to intramuscular fat deposition [28]. The elevated expression of these genes in CX pigs suggests enhanced lipid metabolism and fat deposition capabilities, which align with the higher intramuscular fat content typically observed in indigenous Chinese breeds. Previous studies have demonstrated that increased intramuscular fat content positively correlates with meat tenderness, juiciness, and flavor [29]. Similarly, the up-regulation of ALDH1A3, which participates in the metabolism of aldehydes derived from lipid oxidation, and MGST1, involved in glutathione metabolism and oxidative stress response, may contribute to the development of flavor compounds during meat aging and cooking [30]. These findings are consistent with those reported by Wu et al. (2023), who observed enhanced expression of lipid metabolism genes in Jinhua pigs compared to intensive pig breeds, suggesting a common molecular mechanism underlying the superior meat quality of indigenous Chinese breeds [31].

The enrichment of immune-related functions among up-regulated genes in CX pigs represents an intriguing finding that warrants further investigation. Our GO analysis revealed significant enrichment of terms related to immune response, defense response, regulation of the immune system process, and response to external stimulus among the up-regulated genes in CX pigs. This enhanced expression of immune-related genes may reflect the adaptation of indigenous breeds to more diverse and challenging environmental conditions compared to commercial breeds raised in controlled environments. Several studies have suggested potential connections between immune functions and meat quality traits. For instance, Pothakam et al. (2021) reported that inflammatory responses influence muscle fiber characteristics and intramuscular fat deposition in pigs [32]. The elevated expression of immune-related genes in CX pigs may contribute to their distinctive muscle fiber composition and fat distribution patterns, indirectly affecting meat quality. Additionally, indigenous breeds like CX pigs have been subjected to natural selection for disease resistance over centuries, which may have shaped their immune gene expression profiles. Gu et al. (2023) observed similar patterns of immune gene up-regulation in Tibetan pigs, suggesting that enhanced immune functions may be a common feature of indigenous pig breeds that have evolved under less controlled environmental conditions than commercial breeds [33].

The identification of *PPAR-γ* as a hub gene in our protein–protein interaction network analysis highlights its central role in regulating the transcriptional differences between CX and LAN pigs. *PPAR-γ* is a master regulator of adipocyte differentiation and lipid metabolism, influencing the expression of numerous genes involved in fatty acid uptake, transport, and storage [34,35]. Interestingly, while *PPAR-γ* itself was up-regulated in CX pigs, our KEGG pathway analysis revealed that the *PPAR* signaling pathway was significantly associated with down-regulated genes. This apparent contradiction may reflect complex regulatory mechanisms involving negative feedback loops or differential regulation of specific components within the pathway. The increased expression of *PPAR-γ* in CX pigs likely contributes to their enhanced intramuscular fat deposition and subsequent flavor development. Previous studies have demonstrated that *PPAR-γ* activation promotes intramuscular adipogenesis and improves meat quality in pigs [36,37]. Our findings suggest that differential regulation of *PPAR-γ* and its downstream targets may be a key mechanism underlying the meat quality differences between indigenous and commercial pig breeds.

The enrichment of cytokine and chemokine signaling pathways among up-regulated genes in CX pigs suggests potential roles of inflammatory processes in muscle development and meat quality. Our KEGG pathway analysis revealed significant enrichment of pathways related to viral protein interaction with cytokine and cytokine receptor, the cytokine–cytokine receptor interaction, and the chemokine signaling pathway. Cytokines and chemokines are signaling molecules that regulate inflammatory responses, but they also participate in various physiological processes, including tissue development and remodeling [38]. Ren et al. (2024) reported similar enrichment of inflammatory pathways in the muscle transcriptome of Wuzhishan pigs, suggesting that modulation of inflammatory processes might be a common feature of indigenous pig breeds [39]. Furthermore, certain inflammatory mediators have been shown to influence lipid metabolism, potentially contributing to the development of flavor precursors [40]. These findings highlight the complex interplay between inflammatory signaling, muscle development, and meat quality traits in different pig breeds.

While our study provides valuable insights into the transcriptional differences between CX and LAN pigs, several limitations should be acknowledged. First, our analysis was based on a relatively small sample size of ten individuals per breed, which may limit the statistical power to detect subtle expression differences. Despite this constraint, our findings show strong statistical significance and were validated through qRT-PCR, confirming the robustness of our results within this experimental design. Second, we focused exclusively on the *longissimus dorsi* muscle, while transcriptional differences in other tissues might also contribute to breed-specific characteristics. Different muscles exhibit distinct fiber compositions and metabolic profiles, which could result in tissue-specific gene expression patterns [41]. Third, our study represents a snapshot of gene expression at a single time point, while temporal changes in gene expression during development might provide additional insights into breed differences. Finally, environmental and developmental factors that might influence gene expression were not fully controlled in our study design. Despite these limitations, our study provides a robust foundation for understanding the molecular basis of meat quality differences between indigenous and commercial pig breeds.

In conclusion, our RNA-Seq analysis revealed significant transcriptional differences in the *longissimus dorsi* muscle between Chinese Congjiang Xiang and Landrace pig breeds. The identified DEGs, particularly those involved in lipid metabolism, immune response, and inflammatory signaling, provide insights into the molecular mechanisms underlying the superior meat flavor characteristics associated with indigenous Chinese breeds. The up-regulation of flavor-related genes, including *ELOVL5/6*, *FASN*, *DGAT2*, and *PPAR-γ*, in CX pigs suggests enhanced lipid metabolism and fat deposition capabilities that contribute to their distinctive meat quality traits. Our findings reveal novel functional genes and regulatory networks that may serve as potential molecular markers or targets for genetic improvement programs focusing on enhancing pork quality while maintaining production efficiency. Furthermore, this study contributes to the broader understanding of the genetic architecture governing muscle development in divergent pig breeds and provides valuable resources for future molecular breeding strategies aimed at optimizing meat quality traits in pigs.

## 5. Conclusions

In conclusion, our RNA-Seq analysis revealed significant transcriptional differences between the longissimus dorsi muscles of Chinese Congjiang Xiang and Landrace pig breeds, providing molecular insights into their distinctive meat quality characteristics. The identification of 2459 differentially expressed genes, with up-regulated flavor-associated genes *(ELOVL5/6*, *FASN*, *DGAT2*, *ALDH1A3*, *PPAR-γ*) in Congjiang Xiang pigs, demonstrates the genetic underpinnings of their superior meat flavor. The functional enrichment patterns suggest a potential trade-off between flavor development and muscle structure that differentiates these breeds, with *PPAR-γ* emerging as a central regulatory hub. Future research should focus on three key directions: validation of transcriptional differences through proteomics and metabolomics approaches; investigation of *PPAR-γ* regulatory mechanisms across breeds; and targeted genome editing experiments to establish causal relationships between gene expression and meat quality. Additionally, exploring epigenetic factors would enhance our understanding of meat quality trait regulation. These directions would advance both fundamental knowledge and practical breeding strategies to develop pigs combining indigenous breeds’ meat quality with commercial lines’ production efficiency.

## Figures and Tables

**Table 1:** The top 10 up-regulated DEGs in Longissimus dorsi in CX and LAN pigs.

Gene_Name	Gene_Id	CX	LAN	Log2FC	Adjusted *p*-Value
*AP1S2*	ENSSSCG00045031746	414.59	41.03	3.33	1.00 × 10^−32^
*FCGR3A*	ENSSSCG00045036350	264.93	50.60	2.39	1.05 × 10^−19^
*SLA-DQA*	ENSSSCG00045011704	749.70	105.10	2.83	1.59 × 10^−16^
*CD37*	ENSSSCG00045013461	47.82	8.73	2.45	7.50 × 10^−16^
*EMP2*	ENSSSCG00045000984	477.11	134.64	1.82	1.40 × 10^−14^
*ARMCX2*	ENSSSCG00045011080	47.47	7.32	2.67	1.55 × 10^−14^
*CHPT1*	ENSSSCG00045032049	2151.92	928.29	1.21	2.16 × 10^−14^
*C5AR1*	ENSSSCG00045034745	102.66	14.72	2.80	4.14 × 10^−14^
*DNASE1L3*	ENSSSCG00045035470	49.55	3.38	3.88	2.36 × 10^−13^
*CD53*	ENSSSCG00045035517	183.79	36.60	2.33	5.59 × 10^−13^

**Table 2:** The top 10 down-regulated DEGs in Longissimus dorsi in CX and LAN pigs.

Gene_Name	Gene_Id	CX	LAN	Log2FC	Adjusted *p*-Value
*VPS72*	ENSSSCG00045022741	471.19	1721.26	−1.86	6.47 × 10^−24^
*UBL5*	ENSSSCG00045039454	207.27	1137.93	−2.45	6.84 × 10^−21^
*GDE1*	ENSSSCG00045015707	616.04	1837.79	−1.57	7.50 × 10^−21^
*AQP7*	ENSSSCG00045001378	41.03	217.30	−2.40	1.49 × 10^−19^
*CUL1*	ENSSSCG00045021962	456.96	949.08	−1.05	7.38 × 10^−19^
*DUSP27*	ENSSSCG00045009424	740.94	2549.34	−1.78	1.46 × 10^−18^
*LYSMD2*	ENSSSCG00045031255	103.88	403.52	−1.95	1.26 × 10^−17^
*DDX24*	ENSSSCG00045022939	150.26	411.68	−1.45	2.73 × 10^−17^
*LRRC42*	ENSSSCG00045022986	321.70	889.54	−1.46	3.18 × 10^−17^
*PDZD9*	ENSSSCG00045028818	181.87	1576.16	−3.11	9.77 × 10^−17^

**Table 3:** Relative expression levels of selected differentially expressed genes validated by qRT-PCR.

Gene Name	CX	LAN	Fold Change (CX/LAN)	Log2FC	*p*-Value	RNA-Seq Log2FC	Consistency
*AP1S2*	9.87 ± 1.45	1.06 ± 0.21	9.31	3.22	<0.001	3.33	96.70%
*FCGR3A*	5.64 ± 0.82	1.03 ± 0.18	5.48	2.45	<0.001	2.39	97.50%
*SLA-DQA*	7.42 ± 1.16	1.05 ± 0.22	7.07	2.82	<0.001	2.83	99.60%
*CD37*	5.32 ± 0.94	1.04 ± 0.19	5.12	2.35	<0.001	2.45	95.90%
*EMP2*	3.38 ± 0.56	0.97 ± 0.15	3.48	1.8	<0.001	1.82	98.90%
*VPS72*	0.26 ± 0.04	0.95 ± 0.17	0.27	−1.89	<0.001	−1.86	98.40%
*UBL5*	0.17 ± 0.03	0.96 ± 0.18	0.18	−2.47	<0.001	−2.45	99.20%
*GDE1*	0.34 ± 0.05	0.98 ± 0.20	0.35	−1.51	<0.001	−1.57	96.20%
*AQP7*	0.19 ± 0.04	1.02 ± 0.21	0.19	−2.39	<0.001	−2.40	99.60%
*CUL1*	0.48 ± 0.07	0.99 ± 0.16	0.48	−1.06	<0.001	−1.05	99.00%

## Data Availability

The datasets presented in this article are not readily available due to technical/time limitations. Requests to access the datasets should be directed to the corresponding author.

## Supplementary Materials

The following supporting information can be downloaded at: https://www.mdpi.com/article/10.3390/metabo15070426/s1. Table S1. The details of RT-qPCR primers; Table S2. Overview of RNA Sequencing Data (raw reads, clean reads and mapping rate); Figure S1. Congjiang Xiang pig.
