# Supplementary material for: Transcriptome Profiling Reveals Genetic Basis of Muscle Development and Meat Quality Traits in Chinese Congjiang Xiang and Landrace Pigs
Source: Metabolites. 2025 Jun 22;15(7):426. doi: 10.3390/metabo15070426 (PMC12299553; doi:10.3390/metabo15070426)
Supplement: Supplementary file 1 [file metabolites-15-00426-s001.zip › metabolites-3651356-supplementary.pdf]

Table S1 The details of RT-qPCR primers

Gene name	Forward primer (5'-3')	Reverse primer (5'-3')
AP1S2	GCTGAACCTGACCTCTGTGA	TCTGCAGAGGAAGGTCATCG
FCGR3A	CTCTGCACAGGTGCTCAAAC	GTCTTCCAGGTAGTCGACCA
SLA-DQA	GTCCTGACCACCGTGATCTT	GTAGAGTCGCAGGTCAGCAT
CD37	GCTGTCGGAACTGCTCATCT	ACAGCCTCTGGATGTTGAGG
EMP2	CGTCATCTTCGCCTACCTCA	GAAGACGTAGACGCAGAGGT
VPS72	CTGGACCTGCTGGATGAAGT	CAGACAGTCGGAGTTCAGCA
UBL5	TGAAGGTGGACGGCTACAAG	TCCTCCTCGTAGGAGAACCA
GDE1	GAGGTGGTGAATGGGCAGAT	ACAGAGGCAGGTGTAGTTGG
AQP7	CTCGTGGTAGGCATCAACCT	CCACGTAGATGACCAGGACA
CUL1	AGAAGCAGGACCTCGTGGTA	TGTCCGAGATGGTGTTCTCG
GAPDH	ACATCATCCCTGCTTCTACTGG	CTCGGACGCCTGCTTCAC

Table S2 Overview of RNA Sequencing Data (raw reads, clean reads and mapping rate)

sample	raw_reads	clean_reads	total_map
Lan1	45060506	43886318	91.09%
Lan2	47408450	45788350	91.18%
Lan3	44280184	43672968	90.15%
Lan4	47471914	46647638	90.76%
Lan5	40481638	39676106	90.84%
Lan6	40386450	39590252	90.64%
Lan7	40150042	39416396	91.71%
Lan8	59000094	57016334	92.3%
Lan9	45777180	44748534	89.33%
Lan10	40275636	39130978	91.72%
CX1	42661892	41111216	90.33%
CX2	40823956	39361560	89.44%
CX3	41769290	40066970	88.3%
CX4	41592004	39772906	84.61%
CX5	42292006	40472802	87.39%
CX6	43806900	42148748	87.14%
CX7	41506848	39866240	88.27%
CX8	41895940	40181916	84.21%
CX9	42427602	40501248	89.07%
CX10	45426890	42982948	87.95%

Congjiang Xiang pigs are mainly divided into three lines: Congjiang Zaibian Chuanzi type, Congjiang Jiamian Lingzi type and Congjiang Jiajiuzuiqiao type. Among them, Jiamian Lingzi type Xiang pigs have the largest adult weight, which can reach more than 200 kilograms, while Zaibian Chuanzi type and Jiajiuzuiqiao type Xiang pigs have an adult weight of about 150 kilograms. Zaibian Chuanzi type Xiang pigs are the most numerous and have the best meat quality. They are characterized by red noses, eye circles, Chuanzi-shaped forehead wrinkles, sparse back hair, slightly concave back (obvious back concavity when fed cooked food), short limbs, agile movements, slender tails, tolerance to rough feeding, thin skin, tender meat, sweet taste and good meat taste.

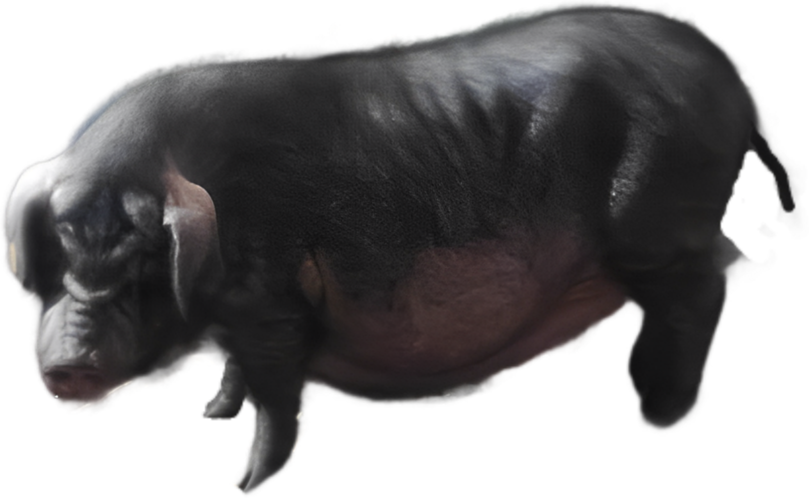

Figure S1. Congjiang Xiang pig
